# Capacity development of researchers involved in guideline development in Malawi, Nigeria and South Africa: A mixed-methods study

**DOI:** 10.4102/phcfm.v18i1.5193

**Published:** 2026-03-31

**Authors:** Retsedisitsoe P. Mazibuko, Willem Odendaal, Sara Cooper, Tamara Kredo, Michael McCaul, Anke Rohwer

**Affiliations:** 1 Division of Epidemiology and Biostatistics, Department of Global Health, Faculty of Medicine and Health Sciences, Stellenbosch University, Cape Town, South Africa; 2 Health Systems Research Unit, South African Medical Research Council, Cape Town, South Africa; 3 Department of Psychiatry, Faculty of Medicine and Health Sciences, Stellenbosch University, Cape Town, South Africa; 4 Cochrane South Africa, South African Medical Research Council, Cape Town, South Africa; 5 School of Family Medicine and Public Health, Faculty of Health Sciences, University of Cape Town, Cape Town, South Africa; 6 Division of Clinical Pharmacology, Faculty of Medicine and Health Sciences, Stellenbosch University, Cape Town, South Africa, South Africa; 7 Centre for Evidence-based Health Care, Division of Epidemiology and Biostatistics, Department of Global Health, Faculty of Medicine and Health Sciences, Stellenbosch University, Cape Town, South Africa

**Keywords:** capacity development, clinical practice guidelines, evidence synthesis skills, researchers, interpersonal skills, sub-Saharan Africa

## Abstract

**Background:**

The Global Evidence, Local Adaptation (GELA) project aimed to build capacity for rigorous clinical practice guideline (CPG) development and evidence-informed decision-making (EDIM) in Malawi, South Africa and Nigeria.

**Aim:**

This study aimed to explore and assess whether and how participating in GELA project activities developed the capacity of GELA researchers in evidence synthesis, guideline development, project management and interpersonal skills.

**Setting:**

GELA researchers were based at academic and research institutions in South Africa, Malawi, Nigeria and Norway.

**Methods:**

We conducted a nested mixed-method study of GELA researchers comprising an online survey and semi-structured interviews. Quantitative data were analysed descriptively, while qualitative data were analysed through framework analysis.

**Results:**

Survey respondents indicated that their confidence in technical skills, as well as project management and interpersonal skills, improved during GELA. Interview results highlighted the importance of both skill sets. Collaboration emerged as a key facilitator of capacity development, while the tension between meeting deliverables and dedicating enough time to capacity development was a key challenge.

**Conclusion:**

The GELA project enabled capacity development in technical, project management and interpersonal skills in novice as well as experienced researchers. The collaborative nature of the project facilitated this iterative process. Planning of capacity development for researchers within a project such as GELA is essential for the success of both capacity development and project deliverables.

**Contribution:**

Our evaluation sheds light on the challenges and facilitators of building capacity of researchers within the context of a multinational project on CPG development.

## Introduction

Evidence-informed decision-making (EIDM) requires the use of the best available evidence to inform effective health decisions.^1^ Production, access to and application of reliable evidence is important globally, but especially in sub-Saharan Africa because of its multiple health challenges and scarce resources.^2,3^ Clinical Practice Guidelines (CPGs) contain recommendations intended to optimise patient care^4^ and should be based on systematic reviews of evidence and assessments of intervention benefits and harms. These recommendations are essential for improving healthcare quality and access, as well as regulating care.^5,6^ Approaches to developing guidelines include developing new guidelines (*de novo* guidelines) and adapting or adopting guidelines to fit the context where they will be used.^7,8^ The GRADE-ADOLOPMENT approach is a methodology that involves combining the advantages of adaptation, adoption and *de novo* to guideline development.^9^ To ensure a rigorous guideline development process, guideline development group (GDG) members need skills to understand and use evidence, while researchers producing the evidence need skills in evidence synthesis. All of these skills culminate towards completing an evidence-to-decision (EtD) table, which provides a transparent record of evidence considered and factors that influence recommendations by GDG members. Evidence-to-decision, also referred to as GRADE EtD, is meant to help groups of panels to use evidence to make decisions in the context of clinical, health systems and public health recommendations in a transparent and structured way.^10^

The Global Evidence, Local Adaptation (GELA) project aimed to increase the capacity of decision-makers and researchers to use evidence to develop locally relevant clinical practice guidelines (CPGs) for newborn and child health in South Africa (SA), Malawi and Nigeria, with additional support from Norwegian partners. The project’s work packages (WPs) align with the guideline development process (WP1, WP2, WP3, and WP4), with a separate WP for capacity building (WP5) and monitoring and evaluation (WP6). Table 1 provides an overview of the GELA project’s work packages and related activities.^11^ Formal training initiatives within WP5 were mostly targeted at the ‘users’ of evidence, in this case, the GDG members within GELA.^5^ However, the ‘producers’ of evidence within GELA, that is, the researchers in each country, also had training needs to support evidence synthesis and guideline development. As CPGs rely on rigorous evidence syntheses, it is essential to ensure that researchers are adequately trained to meet these standards. Within GELA, training activities for researchers were mostly offered when and as needed during the evidence synthesis process. Activities included webinars, journal clubs and short courses.

**TABLE 1:** An overview of global evidence, local adaptation project activities.

Work package	Activities
WP 1: Engaging	Identify and convene country-level guideline panels and guideline steering group committees Conduct rapid baseline assessment of available guidelines and their processes for newborn and child health in South Africa, Malawi and Nigeria Identify national priority topics within child health
WP 2: Evidence synthesis	Find and appraise available CPGs and existing systematic reviews addressing priority topics on newborn and child health, including reviews of effectiveness, acceptability, feasibility and equity impacts and costing Conduct rapid reviews or fast systematic reviews, if needed
WP 3: Decision-making	Complete evidence-to-decision framework for each priority topic Appraise evidence for local country context considering time, place, and setting Convene country-level groups and facilitate consensus to develop recommendations for up to three topics on newborn and child health
WP 4: Dissemination and communication	Produce and test dissemination formats of all recommendations for three audiences: public, patients and healthcare providers Develop and implement country-level knowledge translation (KT) plans Communicate about other aspects of project (e.g. conferences, publications, website and newsletters)
WP 5: Capacity strengthening and sharing	Online course on systematic reviews of effectiveness Online course on systematic reviews of qualitative evidence Online workshops on WHO-like CPG panel simulation Convene quarterly Community of Practice for CPG group members and researchers across countries Bursaries for master’s and post-doctoral students working in evidence-informed policy and practice Bursaries for university short courses on systematic reviews and CPG methods
WP 6: Monitoring and evaluation	Monitor stakeholder engagement activities Evaluate capacity development needs and progress Explore guideline panellists’ experiences with reading and using evidence from qualitative reviews, including their presentation preferences Evaluate stakeholders’ overall views and experiences of the project

*Source*: Adapted from Kredo T, Effa E, Mbeye N, et al. Evaluating the impact of the global evidence, local adaptation (GELA) project for enhancing evidence-informed guideline recommendations for newborn and young child health in three African countries: A mixed-methods protocol. Health Res Policy Syst. 2024;22:114. https://doi.org/10.1186/s12961-024-01189-5.^11^

Note: This table was adopted from the GELA project with permission from the lead author (https://pubmed.ncbi.nlm.nih.gov/39160559/).

GELA, global evidence, local adaptation; WP, work package; CPG, clinical practice guideline.

Capacity development happens through either academic (also referred to as ‘formal capacity development’)^12^ or non-academic training (also referred to as ‘informal capacity development’). Formal capacity development is a structured and planned learning process that is usually supported by academic institutions,^12,13^ commonly resulting in attaining qualifications or certification.^13,14^ Informal capacity development does not lead to a formal qualification^13,14^ and is the unstructured process of learning that often happens as a by-product of problem-solving in the workplace.^12^ It is thus not always possible to identify when and where learning happened, and evaluating informal capacity development can be more complex than evaluating formal capacity development. This article focuses on exploring the impact of capacity development initiatives on GELA researchers (evidence producers), while the capacity development of GELA GDG members (evidence users) is reported elsewhere.^15^

## Research methods and design

### Objectives

We aimed to explore and assess whether and how participating in GELA project activities developed the capacity of GELA project researchers in evidence synthesis, guideline development, project management and interpersonal skills. Specific objectives were to assess researchers’ self-perceived confidence in evidence-informed decision-making (including evidence synthesis and guideline development) before and after GELA, to describe their perceptions and experiences of capacity development within GELA, and identify lessons learned regarding factors such as barriers, enablers and processes for capacity development.

### Study design and participants

We conducted a mixed-method study using quantitative (online survey) and qualitative (semi-structured interviews) data collection across the four project partner countries. Clinical Practice Guideline development processes were implemented in the three African countries. This study fits into the overall evaluation of GELA as outlined in the published protocol.^11^

We (Retsedisitsoe P. Mazibuko and Willem Odendaal) invited all 35 GELA researchers (Malawi *n* = 6, Nigeria *n* = 5, SA *n* = 17 and Norway *n* = 7) to participate in the survey. For the interviews, we invited all South African and Norwegian researchers, comprising WP leads and WP members. Because of time constraints, we purposively sampled Malawian and Nigerian researchers, comprising all WP leads and some members, representing those extensively involved in the project and those less involved.

### Data collection

#### Online survey

We developed a survey (see Online Appendix 1) to assess researchers’ self-perceived confidence in evidence synthesis skills before and after GELA. The survey was based on a tool by Bidonde^16,17^ but adapted to fit the context of this study.

The survey comprised four sections: respondent demographics, core skills (for this study, core skills refer to technical skills), soft skills (project management and interpersonal skills) and capacity development. Core skills consisted of four separate sections: systematic reviews of effectiveness, qualitative evidence synthesis (QES), health economic evidence and methods related to evidence-to-decision processes. Researchers answered only the sections relevant to the research they conducted with the GELA project. Each core skill section consisted of different domains containing specific skills that align with conducting various types of evidence syntheses. Box 1 shows an example of the domain ‘Formulating a review question’ as part of the ‘Systematic review of effectiveness’ section and the specific skills in the domain. Details of all the domains for each section are presented in Online Appendix 1.

BOX 1 Survey example of skills under the domain: ‘Formulating a review question’.

**Formulating a review question:**

- Identifying a gap in evidence and providing a rationale for conducting a systematic review
- Writing a structured research question according to the PICO acronym
- Defining eligibility criteria according to the PICO
- Deciding which study designs to include in the systematic review
- Registering a title and developing a protocol

PICO, population, intervention, comparison, outcome.

Respondents rated their self-perceived baseline confidence in executing different evidence syntheses skills. If they indicated that their skills for a specific domain were built as part of GELA, they also rated their confidence after participating in GELA activities. The confidence ratings for core skills were: ‘No knowledge of this’ (Level 1), ‘I have heard of this topic, but I don’t feel confident to do it (Level 2)’, ‘Slightly confident to do it (Level 3)’ and ‘I have mastered this topic (Level 4)’.

The section on soft (Project management and interpersonal) skills was not separated into domains but listed the specific interpersonal skills, e.g. ‘project coordination meetings’ and ‘chairing project meetings’ (see Online Appendix 1). The confidence ratings for project management and interpersonal skills were: ‘I don’t feel confident’ (Level 1), ‘I feel slightly confident’ (Level 2), ‘I feel moderately confident’ (Level 3) and ‘I feel highly confident’ (Level 4). Finally, we also asked respondents if they developed their skills through participating in the GELA activities, to measure their perceived capacity development.

The survey was designed on REDCap, an electronic data capture tool hosted by the South African Medical Research Council and invitations containing the link to the survey were sent via email. Participation was voluntary, and responses were anonymous; however, there was an option to provide an email address to enable linking survey responses with interview data.

#### Semi-structured interviews

All interviews were conducted virtually on Microsoft Teams except for the five SA member interviews, which were conducted face to face. The WP leads, interviews lasted an average of 52 min, and the WP members’ interviews an average of 41 min. The recordings were transcribed using Autokest.^18^ During the interviews, the interviewers (Retsedisitsoe P. Mazibuko and Willem Odendaal) took observational notes.

To ensure we understood how WPs were set up and what their activities were, we interviewed the WP leads first. The interviews for both WP leads and members focused on their perceptions and experiences of the GELA project more broadly, and whether and how capacity development happened for them. Some questions differed between leads and members, based on their roles within the WP (see Online Appendix 2).

### Data analysis

#### Online survey

Quantitative data obtained from the online survey were analysed using descriptive statistics with Stata 18.^19^ We reported the proportions of GELA researchers who indicated that their skills were developed through GELA activities. For self-perceived confidence levels, we reported the number of responses for each skill per level of confidence (from level 1 confidence, ‘no knowledge’, to level 4 confidence, ‘mastery’) before and after participating in GELA. We assumed that the level of confidence for researchers who had mastered a skill before GELA and thus indicated that they did not build their capacity, remained the same after GELA. We therefore reported on the total number of researchers with level 4 confidence at baseline and after GELA (i.e. the researchers with baseline level 4 confidence plus those with level 4 confidence after participating in GELA activities).

#### Semi-structured interviews

The interview transcripts were analysed with ATLAS.ti,^20^ using framework analysis as proposed by Spencer and Ritchie.^21^ Framework analysis follows a systematic process of data analysis that produces structured outputs of the data.^21,22^ It is flexible and not associated with a particular epistemological, philosophical or theoretical approach.^22^ In addition, framework analysis can be deductive or inductive depending on the research question.^22^ To ensure reliability during analysis, we consulted with the author team to review the analysis process.

In the first step, *Familiarisation*, the lead author started by listening to each recording, followed by her verifying the correctness of the transcriptions and anonymising the data. During this process, she made notes of salient aspects, keeping the objectives and aim of the research in mind. This was followed by the *Thematical Framework development*. Using the study objectives and interview schedules, the lead author and co-interviewer developed the initial code list and organised it into groups and themes, which were uploaded onto ATLAS.ti. They coded 10% of the transcripts independently and thereafter compared their coding. After reaching a satisfactory agreement, the lead author continued with *Indexing*, that is, coding the remaining transcriptions and periodically consulted with the co-interviewer to refine the code list. During the next step, *Charting*, the lead author grouped the codes according to the themes identified in the *Thematical framework development stage*. In the final step, *Mapping*, the lead author reviewed the quotations to confirm additional patterns and interpretations of the data.

### Integration of the quantitative and qualitative data

We used the four principles of Cooke’s framework^23^ of research capacity-building evaluation. This framework is used to evaluate research capacity building at four different structural levels: individual, team, organisational and network and support units using six principles.^23^ However, Principle 1 (skills and confidence building) was the only relevant one to the collected data and was applied to the analysis. We analysed and presented the qualitative and quantitative data separately. We compared and contrasted in the discussion using Principle 1.

### Reflexivity

To ensure trustworthiness, we applied reflexivity, which is the process of acknowledging and critically reflecting throughout the research process upon the researchers’ assumptions and standpoints, and how these may have impacted upon the research.^24^ Anke Rohwer, Michael McCaul, Willem Odendaal, Tamara Kredo and Sara Cooper are GELA researchers. Given their roles within GELA, Anke Rohwer, Michael McCaul, Tamara Kredo and Sara Cooper were participants in this study. Retsedisitsoe P. Mazibuko was a GELA-funded master’s student. The authors are aware that their involvement in the project, in particular WP5, potentially influenced their views and perspectives. At the same time, their intricate knowledge of the project gave valuable insight into the activities and has helped them to interpret the results. Retsedisitsoe P. Mazibuko independently led the data analysis for the interviews and survey to minimise bias during the analysis.

During data collection, the lead author and co-interviewer independently reviewed their interview notes and reflected on how they conducted the interview and thereafter had debriefing sessions.^25^ This sensitised them to unintentionally influencing the interviews through how they asked the questions and responded to interviewees.

During the data analysis, the lead author was aware that her understanding of capacity development and its mechanisms was informed by only the literature on the topic. She continuously reflected and reminded herself to be open to new mechanisms shared by the researchers, and that these might differ from how they are described in the literature.^25^ She was also mindful that her understanding of the literature might affect how she analysed and interpreted the data and constantly reminded herself to stay true to participants’ views and their experiences of capacity development in GELA.

Anke Rohwer, Willem Odendaal and Michael McCaul validated the data interpretations made by the lead author to limit her potential biases.^24^

### Ethical considerations

Ethical clearance to conduct this study was obtained from the Human Research Ethics Committee of the South African Medical Research Council (No. EC015-7/2022). Participation in this study was voluntary. The survey consent was embedded within the survey. If participants consented to the survey, they indicated this by clicking the ‘start’ button in the survey; this was clearly explained in the email that also contained the survey link. We sent consent forms to interview participants, and they signed them to consent to participating in this study and for interviews to be recorded. All identifying data was anonymised. The study data were stored in cloud files that were password-protected.

## Results

We present the demographics for all participants, followed by separate sections on qualitative and quantitative results.

### Participants’ demographics

We invited 35 GELA researchers to complete the survey and received 20 responses (57%) across SA (*n* = 11), Malawi (*n* = 3), Nigeria (*n* = 3) and Norway (*n* = 3).

We conducted 28 interviews comprising nine WP leads and 19 WP members from SA (*n* = 8), Nigeria (*n* = 3), Malawi (*n* = 4) and Norway (*n* = 4). Across the survey and interviews, more than half of the participants were experienced researchers with more than 10 years’ experience and a doctorate (Table 2).

**TABLE 2:** Study participants’ demographics.

Characteristics	Online survey (*N* = 20)		Semi-structured interview (*N* = 28)	
	*n*	%	*n*	%
**Age (years)**				
< 40	7	35	-	-
41–50	8	40	-	-
51–60	4	20	-	-
> 60	1	5	-	-
**Highest level of education**				
Masters	9	45	7	25
Doctoral	11	55	17	61
**Field of work**				
Academia	5	25	8	29
Clinical Epidemiology and Evidence synthesis	1	5	3	10
Health systems and services	3	15	2	7
Public health	11	55	9	32
Knowledge translation, information science, data science and health economy.	-	-	1	4
**Years of experience in their respective field of work**				
5–10	9	45	-	-
11–15	6	30	-	-
> 15	5	25	-	-
**GELA roles**				
Team lead	8	40	9	32
Team member	12	60	19	68

GELA, global evidence, local adaptation.

### Quantitative results

In the survey, 95% (*n* = 19) of the researchers reported that they had conducted evidence synthesis before GELA. Therefore, there were occurrences of no capacity development in some of the core skills (Table 3).

**TABLE 3:** Perceived capacity development of respondents across the four core skills sub-sections.

Skills category	Systematic review of effectiveness (*N* = 13†)		Qualitative evidence synthesis (*N* = 9)		Health economics evaluation (*N* = 6)		Evidence-to-decision (*N* = 12)	
	*n*	%	*n*	%	*n*	%	*n*	%
Formulating a review question	2	15	5	56	3	50	-	-
Searching for studies	4	31	4	44	5	83	-	-
Extracting data	3	23	5	56	3	50	-	-
Critical appraisal	4	31	5	56	4	67	-	-
Summarising study characteristics and preparing for synthesis	3	23	-	-	-	-	-	-
Grading certainty of evidence	3	23	-	-	-	-	-	-
Coding data	-	-	3	33	-	-	-	-
Data analysis and synthesis	3	23	5	56	4	67	-	-
GRADE-CERQual	-	-	7	78	-	-	-	-
Facilitating guideline development structure	-	-	-	-	-	-	9	75
Making judgements about the quality or certainty of evidence	-	-	-	-	-	-	5	42
Transforming evidence to recommendation	-	-	-	-	-	-	8	67

Note: Number of respondents who indicated that their capacity was developed through GELA activities (%).

† , *N* refers to the total number of people who answered the respective sub-section.

Thirteen survey respondents (65%) indicated that they conducted systematic reviews of effectiveness as part of GELA. Baseline confidence was high for the domains: formulating questions, searching for studies, extracting data and assessing the risk of bias. For data synthesis and assessing certainty of evidence, baseline confidence ranged from level 2, level 3, and level 4, but post-GELA activities, the majority of respondents had high confidence in these two domains (Online Appendix 3).

In the survey, 45% (*n* = 9) responded to the QES section, which comprised seven domains. Most respondents reported high baseline confidence in formulating review questions, extracting descriptive data, critical appraisal and coding data domains (detailed in Online Appendix 3). For GRADE-CERQual, 78% (*n* = 7) of respondents indicated that they developed their skills in this domain and reported low baseline confidence but high confidence after participating in GELA activities (see Table 4).

**TABLE 4:** Mastery (level 4 score) of skills: ‘Qualitative evidence synthesis’.

GRADE-CERQual	Qualitative evidence synthesis (*N* = 9)			
	Level 4 confidence before GELA		Level 4 confidence after GELA activities†	
	*n*	%	*n*	%
Understanding the GRADE-CERQual approach to assess the confidence in the evidence	2	22	4	44
Confidence in the evidence: Assessing methodological limitations	3	33	5	56
Confidence in the evidence: Assessing coherence	3	33	5	56
Confidence in the evidence: Assessing adequacy of data	3	33	5	56
Confidence in the evidence: Assessing relevance	3	33	5	56
Generating summary of qualitative findings tables and evidence profiles	3	33	5	56

GELA, global evidence, local adaptation.

† , Total *N* with pre- GELA and post-GELA level 4 confidence.

In the survey, 30% (*n* = 6) of respondents completed the health economics evidence section. In the ‘Searching for studies’ domain, 83% (*n* = 5) of the respondents indicated that they developed their skills through GELA, with post-GELA confidence levels notably higher than baseline confidence for this domain (see Table 5).

**TABLE 5:** Mastery (level 4 score) of skills: ‘Health economics evidence’.

Searching for studies	Health economics evidence (*N* = 6)			
	Level 4 confidence pre-GELA		Level 4 confidence ratings post-GELA†	
	*n*	%	*n*	%
How to develop a search strategy	1	17	4	67
Using controlled vocabulary and text words (MeSH terms, Boolean operators) in a search strategy	1	17	4	67
Using search filters and limits	1	17	4	67
Choosing appropriate databases		17	3	50
Searching electronic databases for evidence	1	17	4	67
Searching for ongoing studies	1	17	3	50
Searching for grey literature (unpublished data)	1	17	3	50
Other search approaches (e.g. hand searching, contacting experts in the field)	1	17	3	50
Using reference management software	4	67	6	100

GELA, global evidence, local adaptation.

† , Total *N* with pre- GELA and post-GELA 4 confidence.

In the survey, 60% (*n* = 12) of researchers responded to the EtD section. Few respondents reported high baseline confidence in the ‘Facilitating guideline development structures and setup’ and in the ‘Transforming evidence to a recommendation’ domains. Notably, the number of researchers who reported high confidence levels in these two domains increased post-GELA activities. Table 6 shows the ‘Facilitating the development of guideline structure and setup’ domain. Online Appendix 3 details the confidence ratings across the different domains.

**TABLE 6:** Mastery (level 4 score) of skill: ‘Evidence-to-decision’.

Facilitating the development of guideline structure and setup	Evidence-to-decision (*N* = 12)			
	Level 4 confidence before GELA		Level 4 confidence after GELA†	
	*n*	%	*n*	%
Overseeing group process and panel composition and conflict of interest management	1	8	4	33
Formulating a healthcare question	3	25	10	83
Overseeing evidence acquisition and interpreting evidence syntheses	3	25	9	75
Identifying priority topics	2	17	6	50
Assessing available clinical practice guidelines	4	33	10	83

GELA, global evidence, local adaptation.

† , Total *N* with pre-GELA and post-GELA level 4 confidence.

In the survey, 70% (*n* = 14) of respondents reported that they strengthened their project management and interpersonal skills. Project management and interpersonal skills that were evaluated were project coordination and management, chairing project meetings, resourcefulness, communication skills, networking, flexibility and adaptation, mentorship and collaboration and working in a multidisciplinary team (Online Appendix 3).

### Qualitative results

#### Theme 1: Technical skills required to produce evidence for guideline development

Participants reported that their respective WPs required specific and different technical skills to achieve the WP objectives (see Table 1).

WP1 focused on scoping priority setting. In this WP, members gained skills in stakeholder mapping and identifying priority topics. For others, GELA strengthened their stakeholder engagement skills.

WP2 focused on finding, appraising and synthesising evidence required to develop clinical guidelines. It required an understanding of three evidence synthesis methods: systematic review of effectiveness, QES and health economics evidence synthesis.

Skills that were strengthened under WP2 included problem-solving in the context of guideline development and evidence synthesis. Some participants mentioned that their critical appraisal skills were strengthened. This included using tools such as the Cochrane Risk of Bias 2 (RoB2) tool, and the AGREE-II tool. Furthermore, they gained skills in assessing the certainty of qualitative evidence using the GRADE CERQual approach. Members were able to strengthen their evidence synthesis skills through conducting QESs and health economic evidence syntheses. Some of the members were also able to learn how systematic reviews are used in guideline development:

‘I think you would need some basic knowledge on qualitative evidence synthesis, and then for the economic evidence you would need some speciality and knowledge to be able to synthesise economic evidence.’ (Participant 12, WP member)

‘I learned how systematic reviews are used in guideline development because what they teach in school … they teach you how to use systematic review, but they don’t quite tap into the part about guideline development.’ (Participant 21, WP member)

WP3 required an understanding of CPG development methods. Experience as a methodologist and that of a systematic reviewer in the context of guideline development was also highlighted as important in this WP. Some of the existing skills that were strengthened included learning about guideline development in a national context, rather than the international context, where various researchers had gained experience prior to joining GELA. Researchers were also able to learn the ADOLOPMENT process of guideline development.

WP4 focused on the dissemination of different formats of CPGs and communication within the project and with external stakeholders. It required knowledge translation skills, such as using evidence to inform decision-making. A participant reported that some of the required technical skills for this WP were the skills to understand study designs and study conduct and the use of evidence to inform decision-making and communicating with decision-makers:

‘For the knowledge translation work, they need to have the knowledge and skill, probably in conducting studies, the study designs and [*someone*] who can critically appraise the results of a study … somebody should be able to synthesise that evidence to make sense and to be able to communicate it that people can understand to make decisions.’ (Participant 20, WP member)

In WP5, some of the required skills included an understanding of capacity development methods, developing workshop content, knowledge of CPG development and evidence synthesis for teaching and learning.

‘So, my role was to support [*removed for blinding*] in teaching and learning aspects. A lot of it was coordination, expertise, and competency that’s required. It is administrative in nature; it’s coordination and administration that’s required, and then the third part is content knowledge around the guidelines in general and evidence synthesis via as a teacher or lecturer in any of these modules or opportunities or community of practices, or seminars.’ (Participant 25, WP member)

Some of the skills that were required in WP6 were protocol writing skills, operationalising of research questions skills and user testing skills. Members were able to strengthen their observation skills through observing guideline development meetings and steering group meetings, specifically, through the iteration of observation tools. An additional skill that was strengthened was developing infographics at a national level.

#### Theme 2: Project management and interpersonal skills were important to achieve project goals

Both technical and soft skills were important to achieve WP objectives. WP leads reported various soft skills that were important to lead their respective WP. Project management, communication and organisation were the skills reported by all leads across all WPs. Work package members were able to build collaboration, planning, project management and time management skills. Both WP members and WP leads emphasised that the GELA project required great collaboration and people skills to be able to carry out their respective roles:

‘I think the people skills. That cuts across all the work packages, having a skill to work and engage with different people with different backgrounds and different personalities.’ (Participant 24, WP member)

#### Theme 3: Collaboration ethos facilitated capacity development

The GELA project, in nature, was a collaborative effort involving partnerships between countries. This collaboration allowed capacity development in the form of learning from others. There were cases of partners from different countries learning from each other, an example of this being partners filling capacity gaps by drawing on the experience of partners in another country with more capacity. Partners would give input on the work performed by other partners to provide guidance, exemplifying learning through collaboration:

‘If our group lacks capacity in a certain area, it gives you a good opportunity to draw from the experiences and the expertise of the other countries within the consortium.’ (Participant 13, WP member)

In addition to learning between countries, there was learning within the countries where individuals within the respective country teams were able to learn from one another. There were instances where researchers from the same country within a WP would meet to discuss areas that others did not understand, and they would take time to share experiences and help their fellow country colleagues understand. It was mentioned that good relationships were important to facilitate learning between individuals:

‘Having good relationships between country teams and members is really essential to making capacity development sustainable, equitable and efficient.’ (Participant 25, WP member)

The collaborative nature of GELA facilitated both informal and formal learning. Examples of formal learning mechanisms within the project were short courses, webinars and workshops. The short courses were provided by Stellenbosch University, one of the GELA partners. This partnership made it easier for the researchers to partake in courses offered. Furthermore, the GELA project provided bursaries for researchers to register for these courses. Another advantage of this collaboration was that the university had courses that were specific to the various required technical skills for CPG development. Some of the webinars were hosted by the Norwegian researchers, while the workshops were hosted by other researchers within the project.

Participants shared their preferences for learning through formal or informal mechanisms. Although the participants had different preferences, some highlighted that formal mechanisms can be more applicable to novice researchers, while those with experience may learn more through informal mechanisms. Many participants suggested that both mechanisms can be complementary:

‘Learning by doing is good in that someone who is in the process they’re seeing how it’s being done while working alongside those who are already knowledgeable about the product or whatever they are working towards. [*Formal learning*] provides the basic competence on which one can then build to be able to do the other part of the process. So, I see them as complementary.’ (Participant 19, WP member)

#### Theme 4: Tension between project deliverables and capacity development

Capacity development deliverables in WP5 of GELA mostly focused on using evidence in the guideline development process. Capacity development addressing gaps in producing evidence (mostly related to WP2) happened within work packages. Because of this, there were reports of challenges in balancing meeting WP deliverables and dedicating time to capacity development. A WP lead thought that there was not enough time to meet both the project deliverables and capacity development. As a result of this, leads had to prioritise meeting the WP deliverables, and capacity development became less of a priority:

‘I think the capacity building could have been optimised if we weren’t in such a rush.’ (Participant 18, WP member)

‘The deliverables were ambitious in terms of what needed to happen in certain time. And there was this tension between getting the job done and making sure people have learned the lesson.’ (Participant 18, WP member)

‘But because the volume of output was so high, and deliverables required so much dedicated time, it kind of left less time for dedicated capacity development.’ (Participant 3, WP lead)

In addition, one WP lead pointed out that there were challenges in implementing the scheduled capacity development activities as detailed in the GELA proposal, and that, some of the planned activities were more challenging to implement in reality than anticipated:

‘Sometimes it’s a challenge because all these proposals are written in advance with the deliverables. But once you’re in the project, you realise, maybe they were not thought of that well, or they’re more challenging than you anticipated, or we’re not able to deliver quite on that.’ (Participant 1, WP Lead)

#### Theme 5: Capacity development was an iterative process

There were reports of the iterative nature of capacity development, and researchers highlighted that the process of learning involved identifying gaps as you progress through the work of the project. WP leads filled these gaps by providing learning resources to their members. One lead took the initiative to consolidate materials that were deemed important to strengthening the skills of the members before the start of the WP activities. In another WP, the leader of a small sub-team started journal clubs to assist with strengthening the skills of the researchers and finding ways to meet the team’s deliverables. Within WP2, there were smaller teams that focused on one of the evidence synthesis methods:

‘Capacity building can be an iterative process. As you’re going along, you identify gaps or people make requests and then those activities can then be set up to train people on those specific area.’ (Participant 3, WP Lead)

‘And all of what we were presenting and engaging with were the steps that we needed to understand and that we needed to go through. So every time we finished a journal club session, we would have made a decision how we are approaching this.’ (Participant 15, WP member)

## Discussion

This mixed-method study explored the capacity development of GELA researchers through an online survey and interviews. Both data sets show that capacity development took place within the GELA project. Capacity development in evidence synthesis was reported in both the interviews and the survey. This was also observed for project management and interpersonal skills. In the interviews, both WP leads and members indicated that they were able to strengthen their skills, and 70% of the survey responses reported capacity development of project management and interpersonal skills.

Many participants had expertise in evidence synthesis and/or CPG development and thus a high baseline confidence in skills. Despite this, we found there was strengthening of capacity within specific evidence synthesis areas and around project management and interpersonal skills. A similar observation was made by Abdullahi et al.,^26^ where the highly skilled senior researchers were able to build their EIDM capacity through training sessions.^26,27^ This emphasises the importance of lifelong, continuous learning for evidence producers, regardless of experience.

Most literature draws attention to the need for technical research skills in low- and middle-income countries.^28^ However, in the GELA project, we found that the researchers not only built their technical skills but also built so-called ‘softer skills’ such as project management and interpersonal skills. Our findings highlight the importance of building interpersonal skills and how these play an essential role in successfully achieving project deliverables. Similarly, Okewole et al.^28^ highlight the importance of developing project management and interpersonal skills for researchers’ career progression, for example, networking skills are needed to interact with other professionals within their fields.^28^

Formal capacity development within the GELA project was supported by academic institutions with existing courses.^3^ This highlights the importance of establishing partnerships with training institutions to facilitate the capacity development of public health researchers serving as evidence producers and methodologists in guideline development activities.^13,29^ This further highlights how collaboration can have an impact on capacity development.

Our findings on formal and informal mechanisms resonate with findings of other studies. Employing different formal and informal mechanisms of learning can increase the chances of successfully building capacity. Izugbara et al.^30^ also reported the importance of introducing different methods of capacity development to support the skill growth of researchers.^30^ The preference for informal learning was also reported by Nurmala,^31^ in this study, informal capacity development of public health professionals belonging to the University of Georgia was assessed. Most of the public health professionals preferred informal learning for reasons such as the ability to deal with challenges immediately, as found in GELA, and expanded discussions with other colleagues, also reported by GELA researchers.^31^ However, this preference does diminish the importance of formal learning as some participants reported that it provides the basic foundation of skills-building.^32^

Our findings have implications for the capacity development of evidence producers involved in projects similar to GELA. Firstly, there should be a deliberate decision to include capacity development for project staff responsible for producing the evidence from the onset of the project. In addition to this decision, capacity development should be planned from the onset of the project, and sufficient resources and time should be allocated to capacity development.

Secondly, we suggest that a blend of formal (workshops, short courses and webinars) and informal learning (experimental learning, meeting discussions and journal clubs) can optimise capacity development.

Finally, there should be measures to cultivate trust and good working relationships between researchers to optimise skills-building.^33^ This was confirmed in the GELA project, with participants sharing how good interpersonal relationships made it easy for skills sharing to happen. In a project similar to GELA, where most interactions happened online, creating opportunities for researchers to interact face to face can be another way of cultivating a good working relationship.

### Strengths and Limitations

This study utilised mixed-methods (interviews and surveys) to provide a broad overview of GELA researchers’ capacity development experiences. This approach allowed for the triangulation of findings, enhancing our understanding. Interviews captured participants’ perceptions and experiences qualitatively, while the survey assessed their confidence levels in various skills both before and after GELA involvement. Comparing these datasets offered a comprehensive view of the skills built.

Several limitations should be noted. The survey data evaluated overall capacity development from GELA activities, not specific learning initiatives. Therefore, we cannot conclude which activities were most effective. A lack of validated tools for evaluating evidence producers’ capacity development led us to adapt a survey tool, which was not validated itself. While survey respondents were mainly skilled researchers, a low response rate prevented us from concluding that all GELA research members were highly skilled. We did not collect information on non-respondents. The survey measured perceived confidence in participants’ ability to perform a skill, which is a subjective, rather than an objective way to measure the development of capacity and did not measure competence (i.e. knowledge, attitudes and skills). Furthermore, we asked respondents to rate their confidence before and after participating in the GELA activities at the same time and not before they engaged in any capacity development activities.

We could not apply Cooke’s framework in the study’s planning, only in the analysis. This likely contributed to only one of its principles being applicable.^23^ In addition, this study only aimed to assess the capacity development of the GELA researchers; we were not involved in the planning of the capacity development of the project. Another factor is that we used the framework to assess capacity development at the individual level, while the framework applies to four structural levels.^23^

Finally, we didn’t link survey responses to interview participants. We found it more appropriate to compare the datasets broadly, and only 10 survey respondents provided their emails, making individual linking impractical.

## Conclusion

The GELA project enabled capacity development in evidence synthesis, guideline development, project management and interpersonal skills in novice as well as experienced researchers. This was an iterative process facilitated by collaboration between project partners. Our findings are based on self-reported measures from a small group of researchers. Planning of capacity development for researchers who are required to produce evidence within a project such as GELA is essential, so that both capacity development and project deliverables can be achieved. There is a need for further research and validation of tools to assess skills needed by evidence producers.
